# High Rates of Undiagnosed Target Organ Damage Among Adults with Elevated Blood Pressure or Diabetes Mellitus in a Community-Based Survey in Lesotho

**DOI:** 10.1007/s44197-023-00158-5

**Published:** 2023-10-26

**Authors:** Emmanuel Firima, Lucia Gonzalez, Moazziz Ali Khan, Molulela Manthabiseng, Mamoronts’sane P. Sematle, Matumaole Bane, Makhebe Khomolishoele, Ikhetheleng Leisa, Lefokotsane Retselisitsoe, Thilo Burkard, Eleonara Seelig, Tristan Lee, Frédérique Chammartin, Ravi Gupta, Bailah Leigh, Maja Weisser, Alain Amstutz, Niklaus Daniel Labhardt

**Affiliations:** 1 Division of Clinical Epidemiology, Department of Clinical Research, University Hospital and University of Basel, Totengässlein 3, 4053 Basel, Switzerland; 2 University of Basel, Basel, Switzerland; 3 Swiss Tropical and Public Health Institute, Basel, Switzerland; 4 SolidarMed, Maseru, Lesotho; 5 Division of Infectious Diseases and Hospital Epidemiology, University Hospital Basel, Basel, Switzerland; 6 Ifakara Health Institute, Ifakara, Tanzania; 7 University of Sierra Leone, Freetown, Sierra Leone; 8 Department of Cardiology, University Hospital Basel, Basel, Switzerland; 9 Medical Outpatient Department and Hypertension Clinic, ESH Hypertension Centre of Excellence, University Hospital Basel, Basel, Switzerland; 10 Norges Blindeforbund, Oslo, Norway; 11 Department of Clinical Research, University Hospital Basel, Basel, Switzerland

**Keywords:** Non-communicable chronic diseases, Arterial hypertension, Diabetes mellitus, Retinopathy, Left ventricular hypertrophy, Renal impairment, Peripheral neuropathy, Lesotho

## Abstract

**Introduction:**

Prevalence of elevated blood pressure (BP) and diabetes mellitus (DM) is increasing in sub-Saharan Africa. Data on target organ damage such as retinopathy, left ventricular hypertrophy (LVH), renal impairment and peripheral neuropathy (PN) among persons with elevated BP and/or DM in sub-Saharan Africa remain scarce.

**Aim:**

To determine at community-level the prevalence of retinopathy, LVH, renal impairment, and PN among adults with elevated BP and/or DM, and assess the association of elevated BP and/or DM with target organ damage in Lesotho.

**Methods:**

During a household-based survey, a sub-sample of adults with elevated BP (≥ 140/90 mmHg) and/or DM (glycosylated hemoglobin ≥ 6.5%), as well as comparators (BP < 140/90 mmHg, HbA1c < 6.5%) were screened for retinopathy, LVH, renal impairment, and PN. We used multivariable logistic regression for inferential analysis.

**Results:**

Out of 6108 participants screened during the survey, 420 with elevated BP only, 80 with DM only, 61 with elevated BP and DM, and 360 comparators were assessed for target organ damage. Among those with elevated BP, and among those with DM with or without elevated BP, prevalence of retinopathy was 34.6% (89/257) and 14.4% (15/104); renal impairment was 45.0% (156/347) and 42.4% (56/132), respectively. Among those with elevated BP, 2.3% (7/300) and 65.7% (224/341) had LVH and left ventricular concentric remodeling, respectively. PN, only assessed among those with DM, was present in 32.6% (42/129). Elevated BP was associated with increased odds of retinopathy (aOR, 19.13; 95% CI, 8.52–42.94; *P* < 0.001) and renal impairment (aOR, 1.80; 95% CI, 1.27–2.55; *P* = 0.001). Presence of both elevated BP and DM was associated with an increased odds of retinopathy (aOR, 16.30; 95%CI, 5.69–46.68; P < 0.001), renal impairment (aOR, 2.55; 95% CI, 1.35–4.81; *P* = 0.004), and PN (aOR, 2.13; 95% CI, 1.04–4.38; *P* = 0.040).

**Conclusion:**

We found a high prevalence of undiagnosed target organ damage among adults with elevated BP and/or DM during community-based screening. These findings emphasize the importance of regular prevention and screening activities in this setting.

**Supplementary Information:**

The online version contains supplementary material available at 10.1007/s44197-023-00158-5.

## Introduction

Over the last decades, prevalence of elevated blood pressure and diabetes mellitus (DM) has been rising in sub-Saharan Africa and is projected to further increase by 2045. [1–3] Both are important risk factors for developing target organ complications like retinopathy, left ventricular hypertrophy (LVH), renal impairment and peripheral neuropathy [4], substantially contributing to the burden of cardiovascular diseases (CVD) in the region [5–8]. However, elevated blood pressure and DM are usually asymptomatic from onset to several years [9], and at diagnosis, individuals may already have developed complications resulting in high morbidity and mortality [10, 11]. At individual level, the presence of target organ damage indicates high to very high cardiovascular risk, and at population level prevalence of target organ damage is an indicator of CVD burden [12–14].

Morbidity and mortality due to target organ damage are rising in low- and middle-income countries as a result of lifestyle change, and the attendant increases in the prevalence of elevated blood pressure, DM and other cardiovascular risk factors which are often under-diagnosed and poorly controlled [15–17]. Few studies investigate the prevalence of target organ damage among persons with elevated blood pressure and DM in sub-Sahara Africa. Published studies report target organ damage prevalence ranging from 32 to 43% in Nigeria [18, 19], 47.5% in Ghana [20], and up to 49% in South Africa [21]. However, most of these studies were conducted in clinic settings where patients visit when symptomatic [20]. In Lesotho, a recent study found a prevalence of elevated blood pressure and DM of 21.6% and 5.3%, respectively [22]. There are, however, no data on the burden of target organ damage in this setting.

The aim of our study is to determine the prevalence of retinopathy, left ventricular hypertrophy, renal impairment, and peripheral neuropathy among individuals found to have elevated blood pressure and/or DM during a household-based survey in Lesotho.

## Methods

### Design and Setting

This study is part of the community-based chronic care Lesotho project (ComBaCaL; www.combacal.org). It is a nested cross-sectional study on prevalence of target organ damage among participants with and without DM and/or elevated blood pressure during a large population-based household survey on non-communicable chronic diseases in two districts in northern Lesotho. For this study, we randomly sampled individuals diagnosed with elevated blood pressure and/or DM, as well as an age- and sex-matched group with neither condition as comparators and screened them for LVH, retinopathy, renal impairment, and peripheral neuropathy.

### Participant Selection

This is a nested study within a population-based survey on prevalence of cardio-vascular risk factors among 6061 adults aged 18 years and above, that was conducted from 1st of November 2021 to 31st of August 2022 in 120 randomly selected village-clusters in Mokhotlong and Butha-Buthe districts [22]. To be eligible for this nested study, participants had to have elevated blood pressure and/or DM, irrespective if they were taking anti-hypertensive or anti-diabetic therapy or not. Further, for persons with high blood pressure, on the day of the survey their blood pressure had to be elevated, defined as a value on the survey day of 140/90 mmHg or above, derived as the mean from the last two out of three measurements. For participants with DM, the glycosylated hemoglobin (HbA1c) value had to be ≥ 6.5% on the day of the survey. Age- and sex-matched survey participants with blood pressure value below 140/90 mmHg, HbA1c below 6.5% and without previous diagnosis of elevated blood pressure and/or DM were enrolled as comparators. As the targeted sample size for this study was not met by the end of the survey, enrolment was extended until November 22^nd^ 2022.

### Procedures and Measurements

Detailed procedures and measurements during the population-based survey have been published [22]. During the survey, randomly selected and consenting household members underwent extensive interviews, clinical and laboratory assessments. If a participant had elevated blood pressure and/or DM, the Open Data Kit (ODK) data collection application flagged the participant as being eligible for this study. We aimed to enroll every second participant with elevated blood pressure, and all with DM. Based on sex, age and location of enrolled participants with elevated blood pressure/DM, the survey team then sampled additional participants with neither condition as comparators.

Blood pressure was measured using the WatchBP® Office ABI automatic blood pressure measurement device manufactured by Microlife® [23]. After 15 min of rest, three measurements were taken with two-minute intervals between measurements. Elevated blood pressure was defined as systolic blood pressure ≥ 140 or diastolic ≥ 90 mmHg, calculated using the average of the second and third readings. Random blood glucose was measured using capillary blood with Accu-Chek® Active glucometer [24]. Those with result ≥ 5.6 mMol/L were further tested for HbA1c levels. HbA1c was initially measured using Jana Care Aina station from 2nd November 2021 to 16th November 2021, and the A1CNow + Professional system thereafter until end of study. DM was defined as a random blood glucose ≥ 5.6 mmol/L and HbA1c ≥ 6.5% and/or taking anti-diabetic medication.

### Assessment of Target Organ Damage

The team assessing for target organ damage was a different team than the one conducting the prevalence survey measurements. For assessment of LVH, renal impairment and peripheral neuropathy, the assessors were not blinded.

#### Retinopathy

Retinopathy was assessed using the Welch Allyn iExaminer System™, a non-mydriatic fundus photographic instrument [25]. Photographs of the optic disc, macula, blood vessels and the peripheral retina were taken from both eyes. These images were then sent to an ophthalmologist for diagnosis and grading according to the International Clinical Diabetic Retinopathy Disease Severity Scale [26] or Scheie classification [27] for diabetic and hypertensive retinopathy, respectively. The ophthalmologist was not aware of elevated blood pressure or DM status of the participant.

#### Left Ventricular Hypertrophy

Left ventricular hypertrophy was assessed only among individuals with elevated blood pressure, and among comparators. Philips Lumify Ultrasonography device [28] was used in a focused echocardiographic procedure. At least 2 loops of parasternal long axis (PLAX) views lasting 3 s each were obtained by trained staff, securely stored as Digital Images and Communications in Medicine (DICOM) files. These were then imported into and analyzed by the Us2.ai image analysis application, an automated, United States Food and Drug Administration (FDA)-cleared, deep learning-based automated program for the analysis of echocardiograms [29]. All measurements were reviewed and approved by one of the co-authors who is an experienced senior cardiologist (TB). Obtained measurements included end-diastolic interventricular septal thickness (IVSd), end-diastolic left ventricular internal diameter (LVIDd) and end-diastolic left ventricular posterior wall thickness (LVPWTd). Left ventricular mass (LVM) was calculated using the formula: LVM = 0.8 × 1.04 × [(IVSTd + LVIDd + LVPWTd)^3^ − (LVIDd)^3^] + 0.6 g. [30] To determine LVH, left ventricular mass index (LVMi_BSA_) was calculated by dividing LVM by body surface area (BSA). Relative wall thickness was calculated using the formula: 2 × LVPWTd/LVIDd. [31]

#### Renal Impairment

A spot urine sample and a venous blood sample were collected for point-of-care determination of urinary albumin-creatinine ratio (ACR; mg/g), and serum creatinine (μmol/l), respectively. ACR was determined using Clinitek® microalbumin analyzer [32]. Blood creatinine was determined using Statsensor® Xpress™ Creatinine Meter [33]. Estimated glomerular filtration rate (eGFR) was calculated electronically using the Chronic Kidney Disease Epidemiology Collaboration (CKD-EPI) equation [34] based on serum creatinine, age, and sex.

#### Peripheral Neuropathy

We screened participants with DM, and comparators for peripheral neuropathy using a 10-g monofilament test. Ten sites on each foot were assessed: the plantar surfaces of the hallux, third and fifth toes; the first, third and fifth metatarsal heads; as well as the medial and lateral aspects of the foot, and the heel; dorsally, the first web space was tested. Presence of sensation to the monofilament on examination was scored 1, absence 0, for a maximum score of 10 and a minimum score of 0.

#### Lifestyle Variables

Additionally, lifestyle variables were obtained, such as if participants ever smoked or consumed alcohol; regularly added salt to already prepared food; consumed a minimum of five servings of fruit or vegetables daily; or engaged in moderate to vigorous physical activity at least 5 days in a week with each daily session lasting at least 30 min.

### Outcome Measures

The outcomes were presence of retinopathy according to the respective diabetic and hypertensive retinopathy grading systems. LVH was present if LVMi_BSA_ was greater than 115 g/m^2^ for men, and greater than 95 g/m^2^ for women [35]. Left ventricular concentric remodeling was defined as absence of left ventricular hypertrophy with relative wall thickness greater than 0.42 [36]. Renal impairment was defined as eGFR below 60 mL/min/1.73m^2^ body surface area and/or urine albumin-creatinine ratio ≥ 30 mg/g [17, 37] according to Kidney Disease Improving Global Outcomes (KDIGO). KDIGO defines chronic kidney disease as eGFR below 60 mL/min/1.73m^2^ body surface area and/or urine albumin-creatinine ratio ≥ 30 mg/g persisting for 3 months or more. However, as our measurements were done once, chronicity could not be established and participants with the mentioned eGFR and urine ACR levels were considered as having renal impairment. Based on urine ACR and eGFR, risk of progression of renal impairment was classified as low risk, moderate risk, high risk, and very high risk using KDIGO risk stratification maps of renal disease progression [38]. Peripheral neuropathy was defined as the presence of a minimum of two insensate areas on 10-g monofilament examination resulting in a score of eight or below [39].

### Statistical Analysis

Descriptive statistics such as mean and standard deviation; and median and interquartile range (IQR) were used for continuous variables, while frequency and percentage were used for categorical variables. We used a logistic regression model adjusted for age, sex, body mass index, physical activity, vegetables and fruit consumption, extra salt consumption, alcohol consumption, smoking, and HIV status to assess the effect of elevated blood pressure and/or DM on the risk of several target organ damages. Missing values were not imputed nor was case-wise deletion done, thus the total number of participants differed across the different models/analyses. KDIGO risk maps [38] were constructed to show prognoses for renal impairment. Statistical analyses were conducted in Stata (16.1, StataCorp LLC, College Station, TX).

### Ethical Considerations

Lesotho’s National Health Research Ethics Committee (NH-REC) approved the study protocol (NH-REC ID 139–2021). Participants provided written informed consent. Reporting of the study follows the Strengthening the Reporting of Observational Studies in Epidemiology (STROBE) reporting guidelines [40].

### Patient and Public Involvement

Representatives of the communities where the prevalence survey was conducted and the public were involved in the design, conduct and dissemination plans of this study.

## Results

**Fig. 1:**
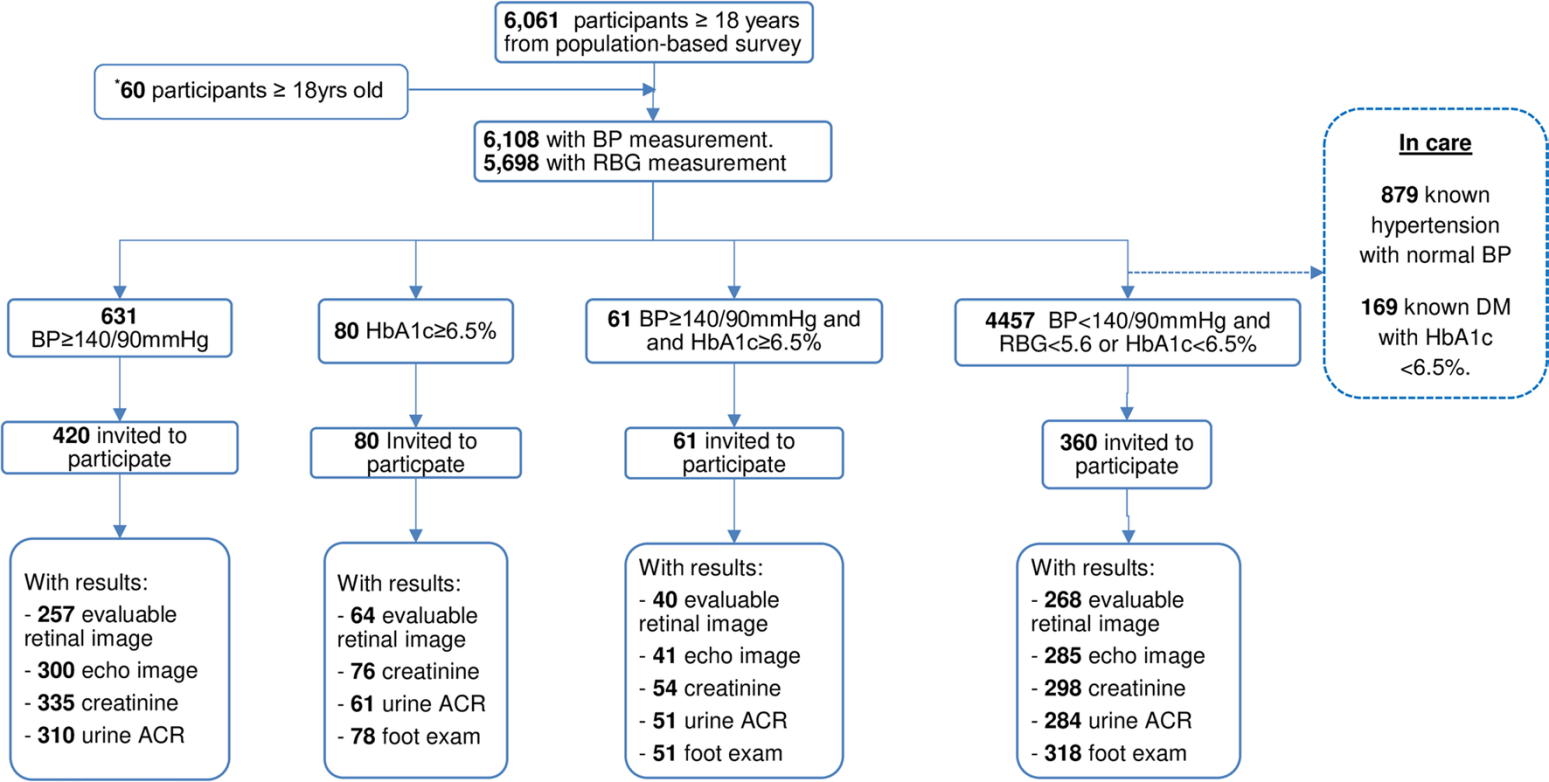
Flow diagram of study population. * Additional data between 01/11/2022 to 30/11/2022; BP: blood pressure, calculated as mean of last 2 of 3 measurements; *RBG* random blood glucose; *HbA1c* glycated hemoglobin; *DM* diabetes mellitus; *ACR* albumin creatinine ratio; echo echocardiography

Figure 1 shows the study flow. After closure of the population-based survey on August 31^st^ 2022, we enrolled an additional 60 participants from the same randomly selected village-clusters, resulting in 6,108 participants with blood pressure and 5698 with random blood glucose results, of which 631 had elevated blood pressure (≥ 140/90 mmHg), 80 had DM with HbA1c ≥ 6.5%, 61 had both conditions, and 4457 had neither condition. Out of these, 420(66.6%), 80(100%), 61(100%) were included into the elevated blood pressure, DM-only, both elevated blood pressure and DM diagnostic groups, respectively. In addition, 360 survey participants matched by sex, age but without elevated blood pressure nor DM were enrolled into the comparator group.

Due to technical reasons, there were missing outcome measurements across elevated blood pressure and/or DM, as well as comparator groups. Thus, the final values included in all analyses differed. The proportion of missing values were similar across the groups for echocardiograms, blood creatinine, urine ACR and foot examination. However, 35.7% (200/561) retinal images were missing among those with elevated blood pressure and/or DM; while 25.6% (92/360) retinal images were missing among comparators. See S1 table in supplemental material.

### Baseline Characteristics

Table 1 displays the participants’ characteristics. Overall, 921 participants were included in this study. The median participant age was 56 years (IQR: 42–67), 556 (60.3%) were female, 176 (19.1%) reported to be living with HIV. Among the 561 participants with either elevated blood pressure or DM, 288 (51.3%) were newly diagnosed during the survey, and 273 (48.7%) were previously diagnosed. Of the 273 with a previous diagnosis, 199 (72.9%) were taking anti-hypertensive and/or anti-diabetic treatment.

**Table 1:** Sociodemographic and cardiovascular disease risk factors among participants by comparator and diagnostic groups

Variable	Elevated BP *n* (%) 420	DM *n* (%) 80	DM/Elevated BP *n* (%) 61	Comparator group *n* (%) 360	Total *n* 921
Age, years, (IQR)	58 (44–70)	54 (38–66)	61 (53–68)	50 (37–65)	56 (42–67)
Age categories					
18–24	14 (3.3)	9 (11.3)	0 (0)	32 (8.9)	55 (6.0)
25–34	34 (8.5)	6 (7.1)	0 (0)	45 (14.5)	85 (9.2)
35–44	58 (13.8)	13 (16.3)	5 (8.2)	63 (17.5)	139 (15.1)
45–54	82 (19.5)	13 (16.3)	12 (19.7)	61 (16.9)	168 (18.2)
55–64	73 (17.4)	17 (21.3)	22 (36.1)	66 (18.3)	178 (19.3)
≥ 65	159 (37.9)	22 (27.5)	22 (36.1)	93 (25.8)	296 (32.1)
Male	179 (42.6)	24 (30.0)	15 (24.6)	148 (41.1)	366 (39.7)
Highest education					
None	52 (12.4)	5 (6.3)	3 (4.9)	44 (12.2)	104 (11.3)
Primary	215 (51.2)	40 (50)	40 (65.6)	172 (47.8)	467 (50.7)
Secondary	120 (28.6)	26 (32.5)	13 (21.3)	116 (32.2)	275 (29.9)
Tertiary	33 (7.9)	8 (10.0)	5 (8.2)	28 (7.8)	74 (8.0)
Mean SBP, mmHg, (SD)	155 (20)	123 (12)	150 (18)	119 (12)	138 (24)
Mean DBP, mmHg, (SD)	94 (13)	76 (8)	86 (11)	75 (8)	85 (14)
Abdominal circumference, cm, (IQR)					
Female	92 (83–103)	97 (85–109)	100 (94–107)	89 (79–98)	92 (82–102)
Male	86 (79–94)	79 (76–95)	100 (90–105)	80 (74–88)	83 (76–92)
BMI, kg/m^2^, (IQR)	26 (23–31)	28 (23–33)	32 (27–36)	24 (21–28)	26 (22–31)
BMI categories					
< 18.5	24 (5.8)	4 (5.0)	1 (1.6)	27 (7.6)	56 (6.1)
18.5–24.9	150 (36.1)	23 (28.8)	9 (14.8)	181 (50.8)	363 (39.8)
25–29.9	117 (28.1)	23 (28.8)	18 (29.5)	75 (21.1)	233 (25.5)
≥ 30	125 (30.1)	30 (37.5)	33 (54.1)	73 (20.5)	261 (28.6)
Blood glucose measures					
RBG, mmol/L, (IQR)	5 (4.6–5.7)	7.9 (6.2–12.8)	10.8 (8.8–16.8)	5 (4.4–5.5)	5.2 (4.6–6.4)
HbA1c, %, (IQR)	5.3 (4.9–5.7)	7.7 (6.9–9.9)	8.2 (7.2–9.5)	5.1 (4.8–5.4)	5.8 (5.1–7.6)
Lifestyle					
Ever smoked	120 (28.6)	13 (16.3)	12 (19.7)	112 (31.2)	257 (28.0)
Alcohol consumption	142 (33.9)	15 (18.8)	10 (16.4)	109 (30.4)	276 (30)
Regular extra salt consumption^a^	58 (13.8)	10 (12.5)	5 (8.2)	45 (12.5)	118 (12.8)
Fruits/vegetables consumption^b^	21 (5.0)	6 (7.5)	0	17 (4.7)	44 (4.8)
Moderate/vigorous physical activity^c^	245 (58.5)	44 (55)	30 (49.2)	231 (64.4)	550 (59.9)
Known HIV infection^d^	67 (16.0)	17 (21.3)	14 (22.9)	78 (21.7)	176 (19.1)
Previous BP/DM diagnosis and treatment					
Known diagnosis	205 (48.8)	19 (23.8)	49 (80.3)	NA	^e^273 (48.7)
On treatment^f^	135 (65.8)	18 (94.7)	46 (93.9)	NA	199 (72.9)

*BP* blood pressure; *DM* diabetes mellitus; *IQR* interquartile range; *SBP* systolic blood pressure; *DBP* diastolic blood pressure; *SD* standard deviation; *RBG* random blood glucose; *HbA1c* glycated hemoglobin; a: 2 missing values (1 elevated BP alone, 1 without either condition), regularly adding salt to already prepared food; b: at least 5 servings daily; c: at least 5 days weekly, each daily session lasting minimum 30 min; d: 2 missing values (1 elevated BP alone, 1 without either condition); e: total is taken for those with elevated BP and/or DM (denominator = 561); f: assessed among participants with known diagnosis

### Prevalence of Target Organ Damage and Progression of Renal Impairment

**Fig. 2:**
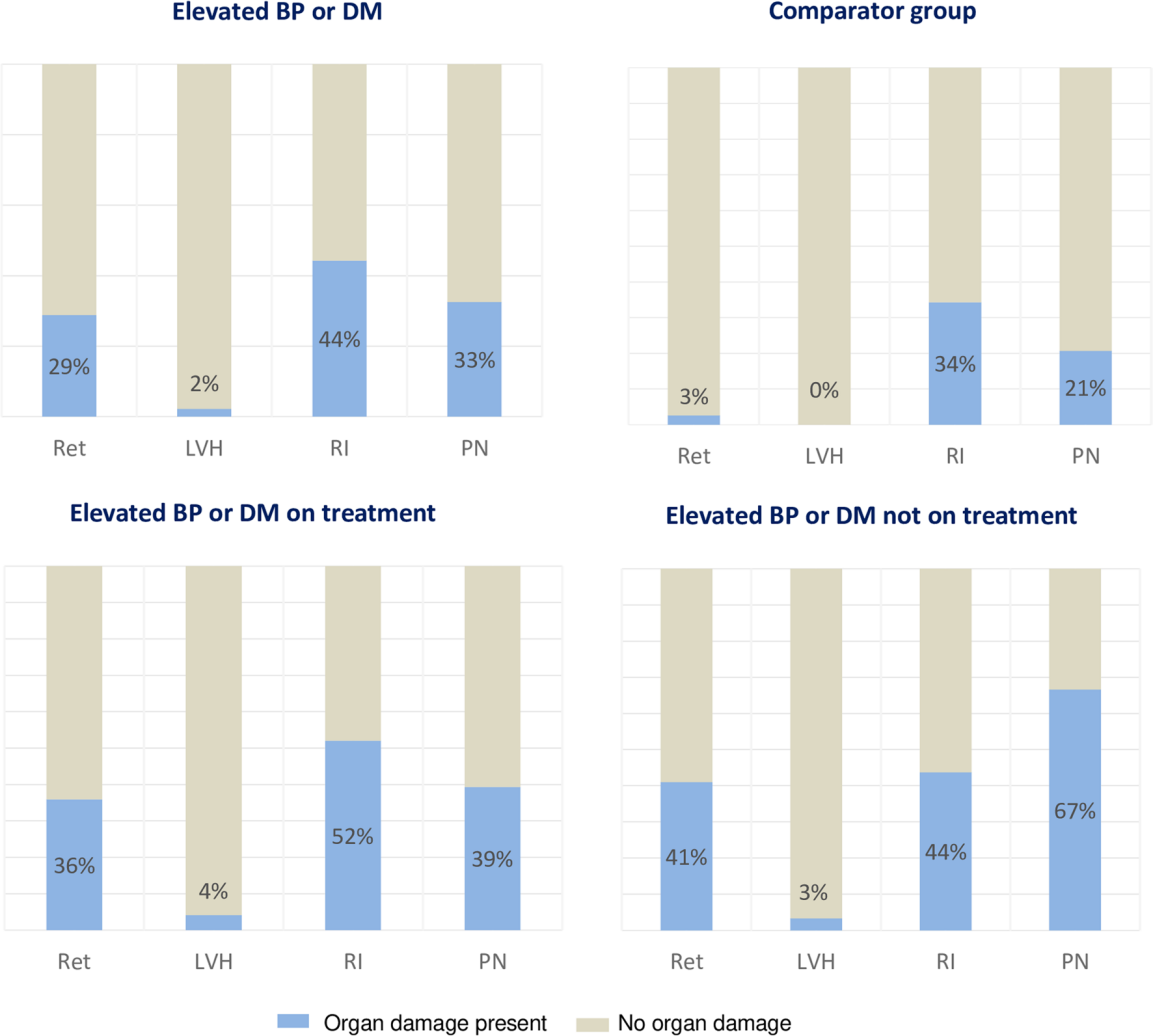
Overall prevalence of target organ damage in different subgroups. More information in supplemental material S3 table. *BP* blood pressure; *DM* diabetes mellitus; *Ret* retinopathy; *LVH* left ventricular hypertrophy; *RI* Renal impairment; *PN* peripheral neuropathy

Table 2 and Fig. 2 display the prevalence of target organ damage in the different groups. Retinopathy was present in 35% (89/257) of participants with elevated blood pressure alone and 14% (15/104) of those with DM with or without elevated blood pressure.

**Table 2:** Prevalence of target organ damage by diagnostic and comparator groups

Outcome	Elevated BP* N*	*n* (%)	DM *N*	*n* (%)	DM and elevated BP *N*	*n* (%)	Comparator group *N*	*n* (%)	Total *N*
Retinopathy	257	89 (34.6)	64	2 (3.1)	40	13 (32.5)	268	7 (2.6)	111/629 (17.7)
Cardiomyopathy	300		NA	NA	41		285		626
Left ventricular concentric remodeling		195 (65.0)				29 (70.7)		131 (46.0)	355 (56.7)
LVH		7 (2.3)				0		0	7 (1.1)
Urine ACR	310		61		51		284		706
< 30 mg/g		192 (61.9)		41 (67.2)		25 (49.0)		205 (72.2)	463 (65.6)
30–300 mg/g		114 (36.8)		18 (29.5)		21 (41.2)		72 (25.4)	225 (31.9)
> 300 mg/g		4 (1.3)		2 (3.3)		5 (9.8)		7 (2.5)	18 (2.6)
eGFR	335		76		54		298		763
≥ 90		163 (48.7)		43 (56.6)		23 (42.6)		175 (58.7)	404 (52.9)
60–89		114 (34.0)		20 (26.3)		16 (29.6)		82 (27.5)	232 (30.4)
45–59		43 (12.8)		6 (7.9)		9 (16.7)		34 (11.4)	92 (12.1)
30–44		10 (3.0)		6 (7.9)		4 (7.4)		5 (1.7)	25 (3.3)
15–29		2 (0.6)		1 (1.3)		1 (1.9)		2 (0.7)	6 (0.8)
< 15		3 (0.9)		0		1 (1.9)		0	4 (0.5)
Renal impairment	347		76		56		303		782
		156 (45)		24 (31.6)		32 (57.1)		104 (34.3)	316 (40.4)
10-g monofilament	NA	NA	78		51		318		447
PN (≥ 2 insensate)				21 (26.9)		21 (41.2)		66 (20.8)	108 (24.2)

*BP* blood pressure; *DM* diabetes mellitus; *LVH* left ventricular hypertrophy (indexed to body surface area); *ACR* albumin creatinine ratio; *eGFR* estimated glomerular filtration rate; *PN* peripheral neuropathy; *NA* not applicable; *N* total number of participants evaluated within each diagnostic/comparator group for each outcome; *n* number with outcome present for each N

Left ventricular hypertrophy, evaluated only among participants with elevated blood pressure and among comparators, was exclusively found in participants with elevated blood pressure, 2.3% (7/300). Left ventricular concentric remodeling was frequent in all three groups, but higher (65.7% [224/341]) for participants with elevated blood pressure with or without DM.

Overall, about one third of participants including comparators presented with albuminuria of ≥ 30 mg/g. Albuminuria was most frequent among participants with elevated blood pressure. The proportion of individuals with reduced eGFR < 60 mL/min per 1.73 m^2^ was highest within the group with both elevated blood pressure and DM: 27.8% (15/54), whereas 17.3% (58/335) individuals with elevated blood pressure alone, 17.1% (13/76) individuals with DM alone, and 13.8% (41/298) comparators had reduced eGFR. Of the 41 participants in the comparator group but with reduced eGFR, 43.9% (18/41) had a known HIV infection. Renal impairment was present in 45% (156/347) participants with elevated blood pressure alone, and 42.4% (56/132) participants with DM with or without elevated blood pressure. Renal impairment was present among 31.6% (24/76) with DM alone, and in 34.3% (104/303) of the comparator group.

**Fig. 3:**
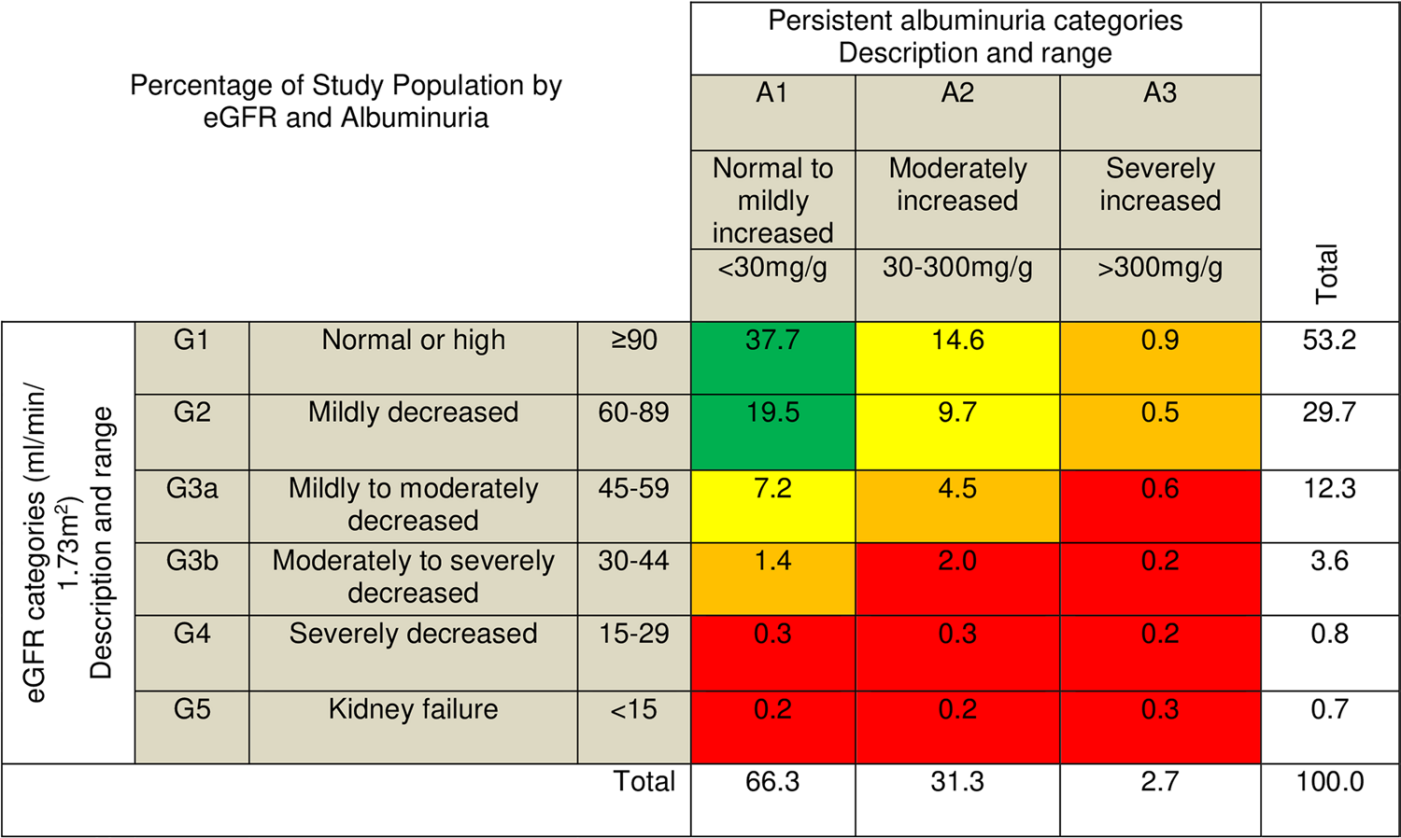
Risk map for progression of renal impairment in the study population. Cells are coded by Kidney Disease: Improving Global Outcomes (KDIGO) [37]. Data are percentages and represent the proportion of all participants within each cell. Risk levels are color-coded as follows: green, low risk (if no other marker of kidney disease); yellow, moderately increased risk; orange, high risk; red, very high risk of progression of renal impairment. eGFR estimated glomerular filtration rate. G eGFR category; A albumin category

**Fig. 4:**
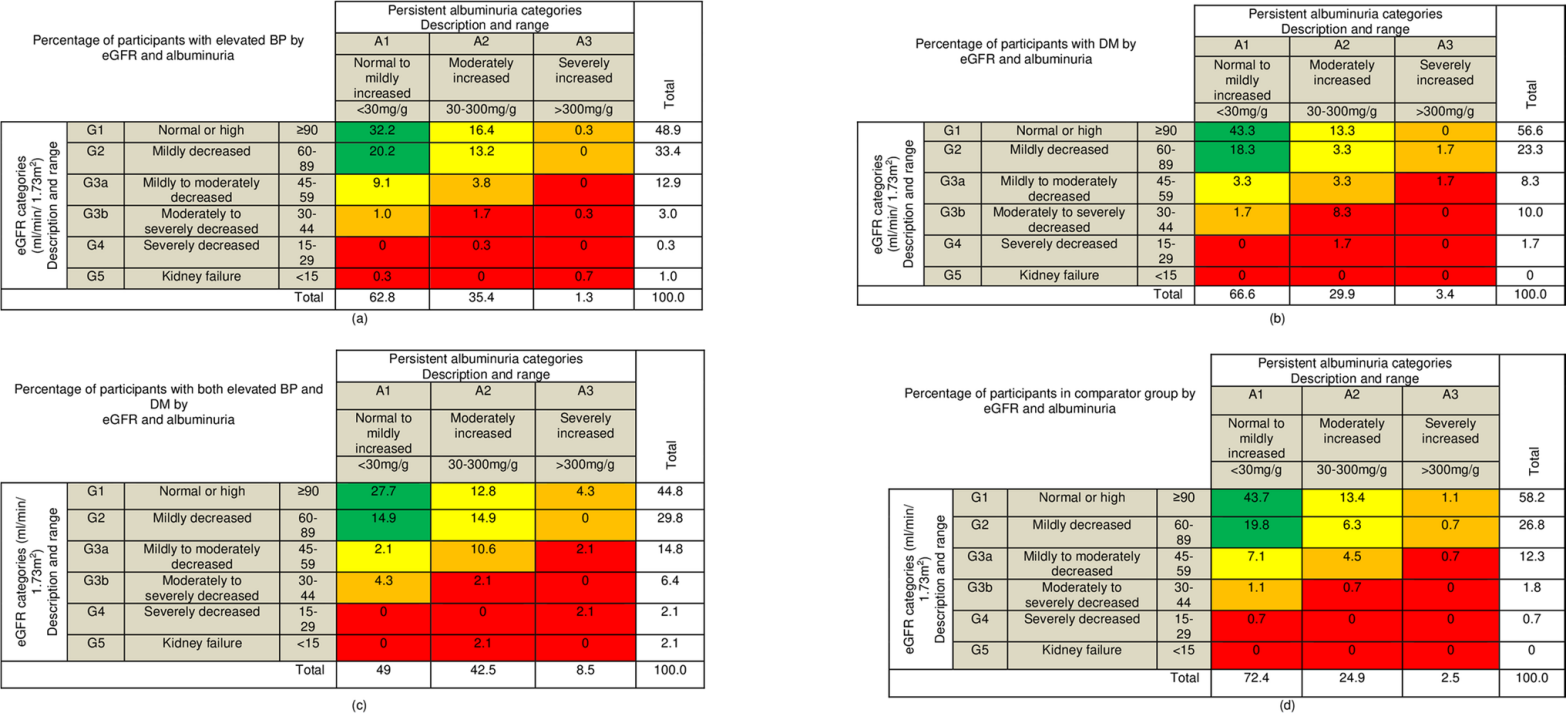
Risk map for progression of renal impairment among **a** elevated BP alone; **b** DM alone; **c** both elevated BP and DM; and **d** comparator group. Cells are coded by Kidney Disease: Improving Global Outcomes (KDIGO) [37]. Data are percentages and represent the proportion of participants in each diagnostic group within each cell. Risk levels are color-coded as follows: green, low risk (if no other marker of kidney disease); yellow, moderately increased risk; orange, high risk; red, very high risk of progression of renal impairment [37]. *eGFR* estimated glomerular filtration rate. G eGFR category; *A* albumin category; *BP* blood pressure; *DM* diabetes mellitus

According to KDIGO risk categories, 12% of the entire study participants had high risk to very high risk of progression of renal impairment. Among participants with elevated blood pressure alone, 8.4% had high to very high risk of progression of renal impairment, while 18.4% with DM alone, 27.6% with both elevated blood pressure and DM, and 9.5% in the comparator group had high to very high risk of progression of renal impairment (Fig. 3 and Fig. 4).

The prevalence of peripheral neuropathy based on two or more insensate sites on monofilament test was 32.6% (42/129) among participants with DM with or without elevated blood pressure. However, among those with both elevated blood pressure and DM, prevalence was 41.2% (21/51); and 26.9% (21/78) among those with DM alone.

Overall, prevalence of target organ damage was higher among participants previously diagnosed vs those newly diagnosed with elevated blood pressure and/or DM. Among participants previously diagnosed with elevated blood pressure or DM, and among those newly diagnosed, retinopathy was 37% (58/156), and 22% (46/205); LVH was 4% (7/178) and 0% (0/163); renal impairment was 50% (118/237) and 39% (94/242); and peripheral neuropathy was 42% (28/67) and 22% (16/73), respectively. (supplemental material S1 figure)

### Target Organ Damage Compared with Participants Without Elevated BP or DM

Table 3 displays multivariable analyses for association of elevated blood pressure and/or DM with target organ damage. Full uni- and multivariable models are presented in supplemental material, S4 to S7 tables. Presence of elevated blood pressure was associated with increasing odds of retinopathy (aOR, 19.13; 95%CI, 8.52–42.94; *P* < 0.001); as was presence of both elevated blood pressure and DM (aOR, 16.30(5.69–46.68); *P* < 0.001). DM alone was, however, not associated with retinopathy. Elevated blood pressure alone was associated with increasing odds of renal impairment (aOR, 1.80; 95%CI, 1.27–2.55; *P* = 0.001). Participants with both elevated blood pressure and DM were about two and a half times more likely to have renal impairment (aOR, 2.55; 95%CI, 1.35–4.81; *P* = 0.004). DM alone was not associated with renal impairment in the study population. Peripheral neuropathy was twice more likely to be present among participants with both elevated blood pressure and DM (aOR, 2.13; 95%CI, 1.04–4.38; *P* = 0.040).

**Table 3:** Multivariable logistic regression on parameter estimates for the different kind of target organ damage assessed in this study *

Parameter	Retinopathy			Left ventricular concentric remodeling		
	*N* (%)	aOR (95 CI	*p*	*N* (%)	aOR (95 CI)	*p*
Comparator group	7/268 (2.6)	1	–	131/285(46.0)	1	–
Elevated BP alone	89/257 (34.6)	19.13 (8.52–42.94)	< 0.001	195/300 (65.0)	1.59(1.10–2.30)	0.013
DM alone	2/64 (3.1)	1.07 (0.22–5.36)	0.930	NA	NA	NA
Both elevated BP and DM	13/40 (32.5)	16.30 (5.69–46.68)	< 0.001	29/41 (70.7)	1.31(0.61–2.83)	0.488

	Renal impairment			PN		
	*N* (%)	aOR (95 CI)	*p*	*N* (%)	aOR (95 CI	*P*
Comparator group	104/303 (34.3)	1	–	66/318 (20.8)	1	–
Elevated BP alone	156/347 (45.0)	1.80 (1.27–2.55)	0.001	NA	NA	NA
DM alone	24/76 (31.6)	0.96 (0.54–1.69)	0.875	21/78 (26.9)	1.50 (0.80–2.83)	0.208
Both elevated BP and DM	32/56 (57.1)	2.55 (1.35–4.81)	0.004	21/51 (41.2)	2.13 (1.04–4.38)	0.040

*adjusted by age, sex, body mass index, physical activity, vegetables and fruit consumption, extra salt consumption, alcohol consumption, smoking, *HIV* more information in supplemental material S4 to S7 tables. *N* number of participants within each disease group with the outcome, see Table 2; *aOR* adjusted odds ratio; *CI* confidence interval; *BP* blood pressure; *DM* diabetes mellitus; *NA* not applicable because target organ damage was not determined within group; *PN* peripheral neuropathy

## Discussion

In this study, on target organ damage among individuals known or newly found to have elevated blood pressure and/or DM compared to individuals with neither condition during a population-based survey in Lesotho, we found high rates of undiagnosed retinopathy, renal impairment and peripheral neuropathy in the entire study population. Unsurprisingly, the odds of target organ damage was substantially higher among individuals with elevated blood pressure and/or DM compared to persons with neither condition. These findings indicate that the study population is at high risk for cardiovascular events [41], progression of renal impairment, and complications due to peripheral neuropathy.

Among study participants with elevated blood pressure, about one out of three had hypertensive retinopathy. Two studies, one conducted in Tanzania, and one in Nigeria among non-diabetic persons with hypertension report a similar prevalence of 32.2% and 37.3%, respectively [42, 43]. In participants with DM, with or without elevated blood pressure, we found a retinopathy prevalence of 14%. Other studies in sub-Saharan Africa conducted among individuals with elevated blood pressure or DM reported higher retinopathy prevalence. They were, however, conducted in clinics, some in tertiary centers with a different study population than the one in our population-based survey [43–45].

Few participants had left ventricular hypertrophy (2%). Left ventricular concentric remodeling was, however, frequent (56%). Reported left ventricular hypertrophy prevalence varies between different studies conducted in sub-Saharan Africa. Using the same echocardiographic approach, one study conducted in a non-probabilistic sample of workers in Angola reported a prevalence of 41% [46], a recent study in Ghana found a prevalence of 4.1% in a random community sample [47]. Whereas we found a low prevalence of left ventricular hypertrophy, over half of the participants had left ventricular concentric remodeling. The evolution of cardiac organ damage is a continuum and risk for remodeling is present in patients with blood pressure below 140/90 mmHg [48]. Left ventricular remodeling is associated with risk of major cardiovascular events and primarily develops due to pressure overload such as occurs in elevated blood pressure [48, 49]. Compared to study participants without elevated blood pressure, left ventricular concentric remodeling was more frequent among individuals with elevated blood pressure.

Contrarily to the findings in a systematic review and meta-analysis on chronic kidney disease (CKD) in sub-Sahara Africa which reported an overall CKD prevalence of 14% [50], and a recent study in Cameroon which reported a prevalence of 11.6% [51], we found a higher prevalence of renal impairment in our study population. Further, applying KDIGO risk maps, we found high proportions of participants at high to very high risk of progression of renal impairment, in line with another study in sub-Saharan Africa [52]. The measurement approach for kidney disease in some of the studies included in the systematic review differed considerably from the definition of renal impairment employed in our study. For example, measuring urine protein was the most frequent method used by studies included in the meta-analysis. Additionally, a CKD prevalence study in six regions of the world reported an overall 36% prevalence among high-risk populations [17], using a KDIGO kidney disease definition similar to the one used in our study. Furthermore, HIV infection and anti-retroviral medications are known risk-factors for kidney disease, and prevalence of CKD up to 48.5% has been reported among individuals with HIV in sub-Sahara Africa [53]. People living with HIV made up 19% of our study population, which may have further contributed to the rather high prevalence of renal impairment in our study.

Our findings on peripheral neuropathy are in line with several studies in the region which reported prevalence of peripheral neuropathy ranging from 15 to 27% [54] among patients with DM. Peripheral neuropathy is one of the commonest complications of DM. While our study is in line with prevalence reports from other sub-Saharan African regions, it also shows high prevalence in the absence of both elevated blood pressure and DM, in a high-burden HIV setting. Similarly to renal impairment, HIV may considerably contribute to the high overall prevalence of peripheral neuropathy in this population.

### Strengths and limitations

An important strength of our study is that the target organ damage screening was conducted in the community as part of a larger household-based survey. Most studies evaluating target organ damage in sub-Sahara Africa have been conducted in hospitals and clinics, which may explain some of the differences in prevalence observed in our study. Secondly, examining target organ damage within each diagnostic subgroup helps display the burden of target organ damage to be expected within these disease groups in Lesotho. Finally, the presence of a matched comparison group without elevated blood pressure or DM improves the estimate of the odds of target organ damage among individuals with elevated blood pressure and/or DM. The study has, however, several limitations. First, causal inference of risk factors and outcomes, as well as temporal associations cannot be established. Secondly, due to the unbalanced missing values for evaluable retinal images between comparators and participants with elevated blood pressure and/or DM, there could potentially be a selection bias. More retinal images where missing among participants with elevated blood pressure and/or DM. These participants generally had poorer image quality which may have been due to ocular and/or retinal conditions. Thus, our result on retinopathy among participants with elevated blood pressure and/or DM could be an underestimate. Thirdly, measurement of blood creatinine and urine albumin were only done once, and abnormal values possibly were transient changes, thus potentially resulting in over-diagnosis of renal impairment. However, for screening purposes at population level, one time measurements of these markers are common and considered acceptable [17]. Fourth, our study setting is a mountainous rural area with poor road network and residents mostly travel on foot. Thus, the resultant foot calluses could have obscured results of monofilament evaluation, and increased the diagnosis of peripheral neuropathy, i.e. in those without DM. Finally, for assessment of LVH, renal impairment and peripheral neuropathy, the assessors were not blinded, introducing a potential observer bias.

## Conclusion

Our data show a high prevalence of undiagnosed target organ damage among persons living with elevated blood pressure and/or DM in Lesotho, indicating a pronounced risk of cardiovascular events or progression of renal impairment. The high rates of target organ damage among adults screened in the community indicate a looming epidemic of cardiovascular disease. These findings emphasize that in addition to the urgently needed prevention and care programs for cardiovascular risk factors such as elevated blood pressure and DM, there is a pressing need to establish regular screening, patient education, and disease management programs for target organ damage in this setting.

### Supplementary Information

Below is the link to the electronic supplementary material.

Supplementary file1 (DOCX 52 KB)

## Data Availability

Data will be shared upon reasonable request to the Head, Division of Clinical Epidemiology, University Hospital Basel, Basel, Switzerland.

## Abbreviations

BP: Blood pressure
DM: Diabetes mellitus
LVH: Left ventricular hypertrophy
PN: Peripheral neuropathy
HbA1c: Glycosylated hemoglobin
aOR: Adjusted odds ratio
CI: Confidence interval
P: P-value
CVD: Cardiovascular diseases
ComBaCaL: Community-based chronic care Lesotho
ODK: Open data kit
PLAX: Parasternal long axis
DICOM: Digital images and communications in medicine
FDA: United States Food and Drug Administration
IVSd: End-diastolic interventricular septal thickness
LVIDd: End-diastolic left ventricular internal diameter
LVPWTd: End-diastolic left ventricular posterior wall thickness
LVM: Left ventricular mass
LVMi_BSA_: Left ventricular mass indexed to body surface area
ACR: Albumin creatinine ratio
eGFR: Estimated glomerular filtration rate
CKD-EPI: Chronic kidney disease epidemiology collaboration
KDIGO: Kidney disease improving global outcomes
IQR: Interquartile range
NH-REC: National Health Research Ethics Committee
STROBE: Strengthening the Reporting of Observational Studies in Epidemiology
SBP: Systolic blood pressure
DBP: Diastolic blood pressure
RBG: Random blood glucose
SD: Standard deviation
HIV: Human immune deficiency virus infection
