# Supplementary material for: High Rates of Undiagnosed Target Organ Damage Among Adults with Elevated Blood Pressure or Diabetes Mellitus in a Community-Based Survey in Lesotho
Source: J Epidemiol Glob Health. 2023 Oct 26;13(4):857–69. doi: 10.1007/s44197-023-00158-5 (PMC10686968; doi:10.1007/s44197-023-00158-5)
Supplement: Supplementary file 1 — Supplementary file1 (DOCX 52 KB) [file 44197_2023_158_MOESM1_ESM.docx]

SUPPLEMENTAL MATERIAL

High rates of undiagnosed target organ damage among adults with elevated blood pressure or diabetes mellitus in a community-based survey in Lesotho

Emmanuel Firima^1,2,3,^, Lucia Gonzalez^1,2,3^, Moazziz Ali Khan^10^, Molulela Manthabiseng^4^, Mamoronts’sane P. Sematle^4^, Matumaole Bane^4^, Makhebe Khomolishoele^4^, Ikhetheleng Leisa^4^, Lefokosane Retselisitsoe^4^, Thilo Burkard^8, 9^, Eleonara Seelig^2,11^, Tristan Lee^1,3^, Frédérique Chammartin^1,2^, Jennifer M. Belus^1,2^, Ravi Gupta^4^, Bailah Leigh^7^, Maja Weisser ^2,5,6^, Alain Amstutz^1,2,#^, Niklaus Daniel Labhardt^1,2,#,*^

1. Division of Clinical Epidemiology, University Hospital Basel, Switzerland
2. University of Basel, Basel, Switzerland
3. Swiss Tropical and Public Health Institute, Basel, Switzerland
4. SolidarMed, Lesotho
5. Division of Infectious Diseases and Hospital Epidemiology, University Hospital, Basel, Switzerland
6. Ifakara Health Institute, Ifakara, Tanzania
7. University of Sierra Leone, Freetown, Sierra Leone.
8. Department of Cardiology, University Hospital Basel, Switzerland.
9. Medical Outpatient Department and Hypertension Clinic, ESH Hypertension Centre of Excellence, University Hospital Basel, Basel, Switzerland
10. Norges Blindeforbund
11. Department of Clinical Research, University Hospital Basel, Switzerland

^#^contributed equally as last authors

*correspondence to: Niklaus Labhardt, Division Clinical Epidemiology, University Hospital and University of Basel, Totgengässlein 3, 4053 Basel, Switzerland, [niklaus.labhardt@usb.ch](mailto:niklaus.labhardt@usb.ch)

S1 Table. Presence or absence of data for outcome measurements among participants with disease vs comparators.

| Outcome measurements | **Elevated BP and or DM**  **N=561** | | **Comparator**  **N=360** | |
| --- | --- | --- | --- | --- |
|  | Available data  n(%) | Missing  n(%) | Available data  n(%) | Missing  n(%) |
| Evaluable retinal images | 361(64.3) | 200(35.7) | 268(74.4) | 92(25.6) |
| Echocardiograms* | 341(70.9) | 127(26.4) | 285(79.2) | 75(20.8) |
| Blood creatinine | 465(82.9) | 96(17.1) | 298(82.8) | 62(17.2) |
| Urine ACR | 422(75.2) | 139(24.8) | 284(78.9) | 76(21.1) |
| Foot exam^#^ | 129(91.5) | 12(8.5) | 318(88.3) | 42(11.7) |

*BP: blood pressure; DM: diabetes mellitus; ACR: albumin creatinine ratio; *echocardiograms were only obtained from those with elevated BP (denominator=481); # foot exam only done among those with DM (denominator =141);*

S2 table. Grading of retinopathy. Total with retinopathy = 111.

| **Grade/grading system** | **Frequency** | **Percent** |
| --- | --- | --- |
| **Hypertensive retinopathy*** | | |
| Stage 1 Scheie | 83 | 76.1 |
| Stage 2 Scheie | 26 | 23.9 |
| Total | 109 | 100 |
| **Diabetic retinopathy^¶^** | | |
| Moderate non-proliferative | 2 | 100 |

**Hypertensive retinopathy graded using Scheie system. ¶ Diabetic retinopathy graded using International Clinical Diabetic Retinopathy Disease Severity Scale.*

S3 table. Overall prevalence of target organ damage among participants with disease and without disease

| **Target organ damage** | **Elevated BP and or DM** | | | **Control** | | |
| --- | --- | --- | --- | --- | --- | --- |
|  | N | Present  n(%) | Absent  n(%) | N | Present  n(%) | Absent  n(%) |
| Retinopathy | 361 | 104(29) | 257(71) | 268 | 7(3) | 261(97) |
| LVH | 341 | 7(2) | 334(98) | 285 | 0 | 285(100) |
| Renal impairment | 479 | 212(44) | 267(56) | 303 | 104(34) | 199(66) |
| PN | 129 | 42(33) | 87(67) | 318 | 66(21) | 252(79) |

*BP: blood pressure; DM: diabetes mellitus; LVH: left ventricular hypertrophy; PN: peripheral neuropathy.*

P=0.002

S1 figure. Prevalence of target organ damage among participants newly diagnosed during survey and those previously known. *PD: Previous diagnosis; ND: New diagnosis; NOD: No organ damage; ODP: Organ damage present*; *LVH: left ventricular hypertrophy; PN= peripheral neuropathy.*

S4 Table. Univariate and multivariable model for association of different parameters with retinopathy. Total screened for retinopathy= 629; with retinopathy= 111.

| **Parameter** | **N(%)** | **OR (95% CI)** | **p** | **aOR(95% CI)** | **p** |
| --- | --- | --- | --- | --- | --- |
| Diagnostic groups |  |  |  |  |  |
| Without elevated BP or DM | 7/268  (2.6) | 1 | - | 1 | - |
| Elevated BP alone | 89/257  (34.6) | 19.75(8.93-43.67) | **<0.001** | 19.13(8.52-42.94) | **<0.001** |
| DM alone | 2/64  (3.1) | 1.20(0.24-5.93) | 0.821 | 1.07(0.22-5.36) | 0.930 |
| Both elevated BP and DM | 13/40  (32.5) | 17.95(6.60-48.83) | **<0.001** | 16.30(5.69-46.68) | **<0.001** |
| Age, years, IQR | 52(42-63) | 1.01(0.99-1.02) | 0.369 | 1.00(0.98-1.01) | 0.767 |
|  |  |  |  |  |  |
| Sex |  |  |  |  |  |
| Female | 64/379  (16.9) | 1 | - | 1 | - |
| Male | 47/251  (18.7) | 1.11(0.74-1.68) | 0.613 | 1.10(0.63-1.91) | 0.733 |
| BMI, Kg/m^2^, IQR | 27(23-32) | 1.03(1.00-1.06) | **0.027** | 1.02(0.98-1.06) | 0.391 |
|  |  |  |  |  |  |
| Vegetables and fruits consumption* |  |  |  |  |  |
| No | 106/597  (17.8) | 1 | - | 1 | - |
| Yes | 5/31  (16.1) | 0.88(0.33-2.35) | 0.799 | 0.91(0.30-2.75) | 0.864 |
| Physical activity^#^ |  |  |  |  |  |
| No | 47/220  (21.4) | 1 | - | 1 | - |
| Yes | 64/408  (15.7) | 0.70(0.46-1.06) | 0.091 | 0.66(0.41-1.06) | 0.085 |
| Smoke |  |  |  |  |  |
| No | 84/466  (18.1) | 1 | - | 1 | - |
| Yes | 27/162  (16.7) | 1.12(0.69-1.79) | 0.652 | 0.99(0.51-1.92) | 0.981 |
| Alcohol |  |  |  |  |  |
| No | 73/446  (16.4) | 1 | - | 1 | - |
| Yes | 38/182  (20.9) | 0.75(0.49-1.17) | 0.204 | 0.94(0.51-1.73) | 0.839 |
| Excess salt consumption^§^ |  |  |  |  |  |
| No | 91/541  (16.8) | 1 | - | 1 | - |
| Yes | 20/87  (23.3) | 1.57(0.92-2.70) | 0.100 | 1.60(0.84-3.04) | 0.149 |
| HIV |  |  |  |  |  |
| No | 81/492  (16.5) | 1 | - | 1 | - |
| Yes | 30/136  (22.1) | 1.42(0.88-2.26) | 0.147 | 1.78(1.03-3.10) | **0.041** |

*OR: odds ratio; aOR: adjusted odds ratio; BP: blood pressure; DM: diabetes mellitus; IQR: interquartile range; BMI: body mass index; HIV: human immune deficiency virus infection; *at least 5 servings daily; # moderate to vigorous exercise at least 5 days in a week; § always or often adds salt or salty sauce to food right before eating. 2 missing observations for each of vegetable and fruit consumption, physical activity, smoking, alcohol, excess salt consumption and HIV.*

S5 Table. Univariate and multivariable model for association of different parameters with left ventricular concentric remodeling. Total screened for left ventricular concentric remodeling= 626; with concentric remodeling= 355.

| **Variable** | **N(%)** | **OR (95% CI)** | **p** | **aOR(95% CI)** | **p** |
| --- | --- | --- | --- | --- | --- |
| Diagnostic groups |  |  |  |  |  |
| Without elevated BP or DM | 131/285  (46.0) | 1 | - | 1 | - |
| Elevated BP alone | 195/300  (65.0) | 2.18(1.57-3.04) | **<0.001** | 1.59(1.10-2.30) | **0.013** |
| DM alone | NA | NA | NA | NA | NA |
| Both elevated BP and DM | 29/41  (70.7) | 2.84(1.39-5.79) | **0.004** | 1.31(0.61-2.83) | 0.488 |
| Age, years, IQR | 61(47-71) | 1.04(1.03-1.05) | **<0.001** | 1.04(1.03-1.05) | **<0.001** |
|  |  |  |  |  |  |
| Sex |  |  |  |  |  |
| Female | 223/373  (59.8) | 1 | - | 1 | - |
| Male | 132/253  (52.2) | 0.73(0.53-1.01) | 0.060 | 1.05(0.70-1.58) | 0.806 |
| BMI, Kg/m^2^, IQR | 26.4(22.7-31.3) | 1.07(1.04-1.10) | **<0.001** | 1.06(1.03-1.10) | **<0.001** |
|  |  |  |  |  |  |
| Vegetables and fruits consumption* |  |  |  |  |  |
| No | 342/594  (57.6) | 1 | - | 1 | - |
| Yes | 12/30  (40.0) | 0.49(0.23-1.04) | 0.063 | 0.48(0.22-1.09) | 0.080 |
| Physical activity^#^ |  |  |  |  |  |
| No | 146/241  (60.6) | 1 | - | 1 | - |
| Yes | 208/383  (54.3) | 0.77(0.56-1.07) | 0.124 | 0.95(0.66-1.37) | 0.775 |
| Smoke |  |  |  |  |  |
| No | 256/440  (58.2) | 1 | - | 1 | - |
| Yes | 98/184  (53.3) | 1.22(0.86-1.73) | 0.258 | 0.93(0.60-1.44) | 0.741 |
| Alcohol |  |  |  |  |  |
| No | 247/422  (58.5) | 1 | - | 1 | - |
| Yes | 107/202  (53.0) | 1.25(0.89-1.76) | 0.190 | 1.07(0.70-1.64) | 0.763 |
| Excess salt consumption^§^ |  |  |  |  |  |
| No | 309/539  (57.3) | 1 | - | 1 | - |
| Yes | 45/85  (52.9) | 0.84(0.53-1.32) | 0.448 | 1.05(0.63-1.75) | 0.846 |
| HIV |  |  |  |  |  |
| No | 294/511  (57.5) | 1 | - | 1 | - |
| Yes | 60/113  (53.1) | 0.84(0.56-1.26) | 0.389 | 0.99(0.64-1.54) | 0.975 |

*OR: odds ratio; aOR: adjusted odds ratio; BP: blood pressure; DM: diabetes mellitus; IQR: interquartile range; BMI: body mass index; HIV: human immune deficiency virus infection; *at least 5 servings daily; # moderate to vigorous exercise at least 5 days in a week; § always or often adds salt or salty sauce to food right before eating. 2 missing observations for each of vegetable and fruit consumption, physical activity, smoking, alcohol, excess salt consumption and HIV.*

S6 Table. Univariate and multivariable model for association of different parameters with renal impairment. Total screened for renal impairment= 782; with renal impairment= 316.

| Variable | N(%) | OR (95% CI) | p | aOR(95% CI) | p |
| --- | --- | --- | --- | --- | --- |
| Diagnostic groups |  |  |  |  |  |
| Without elevated BP or DM | 104/303  (34.3) | 1 | - | 1 | - |
| Elevated BP alone | 156/347  (45.0) | 1.56(1.14-2.15) | **0.006** | 1.80(1.27-2.55) | **0.001** |
| DM alone | 24/76  (31.6) | 0.88(0.52-1.51) | 0.651 | 0.96(0.54-1.69) | 0.875 |
| Both elevated BP and DM | 32/56  (57.1) | 2.55(1.43-4.56) | **0.002** | 2.55(1.35-4.81) | **0.004** |
| Age, years, IQR | 61(46-72) | 1.02(1.01-1.03) | **<0.001** | 1.02(1.01-1.03) | **<0.001** |
|  |  |  |  |  |  |
| Sex |  |  |  |  |  |
| Female | 213/470  (45.3) | 1 | - | 1 | - |
| Male | 103/313  (32.9) | 0.59(0.44-0.80) | **0.001** | 0.55(0.38-0.78) | **0.001** |
| BMI, Kg/m^2^, IQR | 25.5(21.9-30.4) | 0.99(0.97-1.01) | 0.535 | 0.97(0.94-0.99) | **0.015** |
|  |  |  |  |  |  |
| Vegetables and fruits consumption* |  |  |  |  |  |
| No | 303/743  (40.8) | 1 | - | 1 | - |
| Yes | 13/38  (34.2) | 0.76(0.38-1.50) | 0.422 | 0.90(0.44-1.86) | 0.781 |
| Physical activity^#^ |  |  |  |  |  |
| No | 146/310  (47.1) | 1 | - | 1 | - |
| Yes | 170/471  (36.1) | 0.63(0.47-0.85) | **0.002** | 0.71(0.52-0.97) | **0.030** |
| Smoke |  |  |  |  |  |
| No | 219/559  (39.2) | 1 | - | 1 | - |
| Yes | 97/222  (43.7) | 0.83(0.61-1.14) | 0.246 | 0.67(0.45-0.98) | **0.041** |
| Alcohol |  |  |  |  |  |
| No | 231/551  (41.9) | 1 | - | 1 | - |
| Yes | 85/230  (37.0) | 1.23(0.90-1.69) | 0.198 | 1.18(0.79-1.75) | 0.419 |
| Excess salt consumption^§^ |  |  |  |  |  |
| No | 291/691  (42.1) | 1 | - | 1 | - |
| Yes | 25/89  (28.1) | 0.53(0.33-0.86) | **0.010** | 0.60(0.36-1.01) | 0.053 |
| HIV |  |  |  |  |  |
| No | 238/633  (37.6) | 1 | - | 1 | - |
| Yes | 78/147  (53.1) | 1.88(1.31-2.70) | **0.001** | 2.07(1.41-3.04) | **<0.001** |

*OR: odds ratio; aOR: adjusted odds ratio; BP: blood pressure; DM: diabetes mellitus; IQR: interquartile range; BMI: body mass index; HIV: human immune deficiency virus infection; *at least 5 servings daily; # moderate to vigorous exercise at least 5 days in a week; § always or often adds salt or salty sauce to food right before eating. 2 missing observations for each of vegetable and fruit consumption, physical activity, smoking, alcohol, excess salt consumption and HIV.*

S7 Table. Univariate and multivariable model for association of different parameters with peripheral neuropathy. Total screened for PN= 447; with PN= 108.

| Variable | N(%) | OR (95% CI) | p | aOR(95% CI) | p |
| --- | --- | --- | --- | --- | --- |
| Diagnostic groups |  |  |  |  |  |
| Without elevated BP or DM | 66/318  (20.8) | 1 | - | 1 | - |
| Elevated BP alone | NA | NA | NA | NA | NA |
| DM alone | 21/78  (26.9) | 1.41(0.80-2.48) | 0.240 | 1.50(0.80-2.83) | 0.208 |
| Both elevated BP and DM | 21/51  (41.2) | 2.67(1.44-4.97) | **0.002** | 2.13(1.04-4.38) | **0.040** |
| Age, years, IQR | 63(52-75) | 1.05(1.03-1.06) | **<0.001** | 1.04(1.03-1.06) | **<0.001** |
|  |  |  |  |  |  |
| Sex |  |  |  |  |  |
| Female | 68/278  (24.5) | 1 | - | 1 | - |
| Male | 40/169  (23.7) | 0.96(0.61-1.50) | 0.850 | 1.19(0.68-2.06) | 0.543 |
| BMI, Kg/m^2^, IQR | 25.1(20.9-30.1) | 1.00(0.97-1.04) | 0.844 | 0.99(0.94-1.03) | 0.527 |
|  |  |  |  |  |  |
| Vegetables and fruits consumption* |  |  |  |  |  |
| No | 104/426  (24.4) | 1 | - | 1 | - |
| Yes | 4/20  (20.0) | 0.77(0.25-2.37) | 0.653 | 0.97(0.29-3.22) | 0.957 |
| Physical activity^#^ |  |  |  |  |  |
| No | 60/178  (33.7) | 1 | - | 1 | - |
| Yes | 48/268  (17.9) | 0.43(0.28-0.67) | **<0.001** | 0.55(0.34-0.89) | **0.014** |
| Smoke |  |  |  |  |  |
| No | 78/319  (24.5) | 1 | - | 1 | - |
| Yes | 30/127  (23.6) | 1.05(0.65-1.70) | 0.854 | 1.02(0.57-1.82) | 0.948 |
| Alcohol |  |  |  |  |  |
| No | 84/333  (25.2) | 1 | - | 1 | - |
| Yes | 24/113  (21.2) | 1.25(0.75-2.09) | 0.393 | 1.10(0.58-2.11) | 0.763 |
| Excess salt consumption^§^ |  |  |  |  |  |
| No | 98/399  (24.6) | 1 | - | 1 | - |
| Yes | 10/47  (21.3) | 0.83(0.40-1.73) | 0.619 | 1.02(0.44-2.35) | 0.962 |
| HIV |  |  |  |  |  |
| No | 83/348  (23.9) | 1 | - | 1 | - |
| Yes | 25/98  (25.5) | 1.09(0.65-1.83) | 0.735 | 1.23(0.71-2.15) | 0.460 |

*OR: odds ratio; aOR: adjusted odds ratio; BP: blood pressure; DM: diabetes mellitus; IQR: interquartile range; BMI: body mass index; HIV: human immune deficiency virus infection; *at least 5 servings daily; # moderate to vigorous exercise at least 5 days in a week; § always or often adds salt or salty sauce to food right before eating. 1 missing observation for each of vegetable and fruit consumption, physical activity, smoking, alcohol, excess salt consumption and HIV.*
